# The supporting effects of high luminous conditions on grade 3 oral reading fluency scores

**DOI:** 10.1186/2193-1801-3-53

**Published:** 2014-01-25

**Authors:** Michael S Mott, Daniel H Robinson, Thea H Williams-Black, Susan S McClelland

**Affiliations:** The University of Mississippi, Oxford, MS USA; Colorado State University, Oxford, MS USA; Advanced Education Center, The University of Mississippi, 1918 Briar Ridge Road, 38804 Oxford, MS USA

## Abstract

The universality of the impact of daylight is a common thread that defines humanity. Day light affects us in a variety of ways –visually, psychologically and biologically. Artificial lighting research has explored ways in which artificial lighting may substitute for daylight and enhance human health and wellbeing. Recently, a study by Mott et al. 2011 found that the usage of high intensity, yet glare free lighting, (referred to as Focus light setting) during reading instruction increased grade 3 students’ oral reading fluency (ORF) scores, a key index of reading comprehension. The current study further explored the effect of Focus lighting during literacy instruction with at-risk grade 3 students (n = 172). Over the course of an academic year, the Focus lighting students increased their ORF scores at a greater rate than did the Normal lighting students. These findings, in combination with earlier lighting research, suggest that artificial lighting plays a key role in helping to create an effective learning environment to ensure children reach their full potential. More systematic research, however, is needed to understand the mechanisms by which artificial lighting may contribute to the learning environment: visually, biologically and/or psychologically.

## Background

During childhood, children develop essential lifelong skills that will support them throughout their adult lives. While educational research has provided valuable insights into the importance of various aspects of learning environments, such as learning tasks and materials, time on task, teachers’ instructional behavior, and the relationship between teacher and student (Marzano 2007), systematic empirical research into the influence of physical aspects of students’ learning environment remains scarce. Air quality and temperature (Fisk et al. 2003; Shendell, et al.^2004^; Wargocki et al. 2005), noise (Earthman 2004; Evans & Stecker 2004; Higgins et al. 2005), music (Pawananon, et al. 2009), plants (Bringslimark et al. 2009), color (Engelbrecht 2003) and lighting (Dunn et al. 1985; Boyce et al. 2003) are all reported to influence children’s school performance and well-being.

In this paper, the researchers focused on the effects artificial lighting can have on the learning environment of grade 3 at-risk school children in a Mid-south regional public elementary school. Human function is enabled and directed by light (Boyce et al. 2003) as light enables visual abilities and reduces the chance of myopia (Grangaard 1995); systematizes bodily processes (Dijk & Cajochen 1997). Moreover, light is reported to be critical for vitamin D production (Veitch & McColl 2001).

The importance of daylight for mankind and knowing that nowadays children spend around 85cb of their time indoors (Boyce et al. 2003; Dijk & Cajochen 1997) suggests that creating a ‘healthy’ learning environment will be key to children’s development and ensuring that their potential is unlocked. So just exactly what roles can artificial light play in creating an optimum-learning environment?

Children’s school functioning and performance has been reported to benefit from natural daylight (Boyce et al. 2003) and artificial lighting (Boyce et al. 2003; Govén et al. 2010). Windows provide natural daylight, as well as a view. Both factors are relevant to school performance.

Next to daylight, artificial light is reported to support children’s visual, cognitive and behavioral skills. Pre-schoolers are often unable to suppress task-irrelevant thoughts and cannot sustain attention for very long, and,as a result, teachers tend to switch classroom activities every 15-20 minutes.

Bright and/or blue enriched lighting has proven to help maintain a better mood during the day (Govén et al. 2010; Govén et al. 2011). This in turn enhances children’s ability to concentrate, especially if their biological clock is geared towards sleep during the first few hours as a result of going to bed late, which is common among adolescents. In addition to the development of children’s cognitive and behavioral skills, lighting can support the development of children’s visual skills by helping to improve visibility and to support the child’s ability to track material in books visually (Bringslimark et al. 2009; Earthman 2004; Engelbrecht 2003; Evans & Stecker 2004; Higgins et al. 2005; Pawananon et al. 2009). Field studies in schools using high intensity and color temperatures in the order of daylight referred to as a “Focus” setting revealed that the usage of focus setting enables children to read with increased speed (Mott et al. 2011). Mott et al. (2011) confirmed findings by measuring all three components of oral reading fluency (ORF): speed, accuracy and expression, and not just reading speed.

The current study seeks to examine the Focus lighting effect on ORF when studied in the field rather than finding out the mechanism. ORF is a vital element of competent reading. The Mott et al. 2011 study compared ORF reading scores measured by the standardized AIMSWeb instrument with grade 3 students from middle-income backgrounds (based upon a zero percent free/reduced lunch) and found a Focus lighting effect for improving student ORF. The hypothesis is that by offering lighting conditions that support the children biologically, psychologically and/or visually during the literacy lessons, the uptake of the information will be promoted and as a consequence children will perform better during the examination of ORF. The study has been set up as a quasi-experimental study to demonstrate the effect of the usage of dynamic lighting during the literacy instruction. The current study (n = 88) u examined effects for low SES, at-risk students, and utilized a different norm referenced ORF measure (STAR versus AIMS Web). The different test was due to a change initiated by the local education district that preferred the reporting-out features of STAR versus AIMSWeb.

## Method

A system for dynamic lighting of classrooms was designed to support the rhythm of activity in the classroom with four different lighting settings. The teacher is able to select the most appropriate setting via a five-button, wall-mounted control panel located in the classroom. The system has three preset lighting settings:

1. Focus setting. This setting aids concentration during challenging tasks, such as instruction, exams and tests. The average horizontal illuminance measured at desk level is 1000 lx with a CCT of 6500 K (a bright white light).
2. Calm setting. This setting brings a relaxing ambience to support independent and collaborative learning. The average horizontal illuminance measured at desk level is 300 lx with a CCT of 2900 K (white light with a warm, red colour tone).
3. Normal setting. This lighting setting is used for regular classroom activities. The average horizontal illuminance measured at desk level is 500 lx, and the CCT is 3500 (standard white light as commonly used in indoor workplaces).

The settings were created by colour-mixing the light output from a surface-mounted luminaire “consisting of a Modified Softrace with three T5HO lamps: two 17000 K Activiva Active and one 2700 K, with one 1-lamp DALI ballast, one 2-lamp DALI ballast, and one DMBC320–DALI-NA controller. Readings were taken in the center, front, and corners of the room, and the average maintained light levels were computed based on those various readings. The original lighting in the classrooms (lens troffer 2 by 4 two-lamp T8 fluorescent fixtures) which offered 500 lux at the desk with a CCT of 3500 K still remains in the control classrooms.

### Design and participants

A quasi-experimental research design was used to test the hypothesis that by offering high intensity lighting conditions during the literacy lessons, the uptake of the information will be promoted and as a consequence, children will perform better during the examination of ORF. The chosen quasi-experimental design is similar to that used by Mott et al. (2011): 88 at-risk grade 3 students^a^ (in 6 classrooms in a public elementary school in the mid-South region of the United States were randomly assigned to either the experimental group experiencing “Focus” or the control group experiencing “Normal” classroom light conditions. The school administrative method for establishing the four groups prior to the school year (start of research) was accomplished randomly but with students with learning disabilities excluded. The classrooms randomly assigned to become part of the experimental group were equipped with the dynamic light system in 3 classrooms before the start of the academic year. To avoid Hawthorne effects, and strive for equivalency of groups, teachers were instructed to not discuss the type of lighting in their classroom with their students.

All participants received daily ORF instruction for a period of 45 minutes with the same instructional methodology across all classrooms. A literacy coach and the principal conducted weekly classroom checks to monitor and provide consistent feedback to all teachers to ensure that the mandated curriculum and instruction was applied. Half of the classrooms activated the Focus setting during ORF instruction and the other half of the classrooms utilized Normal lighting and all testing was performed in Normal lighting conditions. Instructional equivalence was additionally increased via teacher ORF training completed prior to the start of the school year. The Focus lighting condition was activated during literacy instructions compared to Normal lighting utilized in control classrooms Table 1 demonstrates a typical day routine.

**Table 1:** **Means (and standard deviations) oral reading fluency scores by lighting condition at the three different times**

	n	Time 1	Time 2	Time 3
Focus lighting	43	65.05 (18.08)	72.24 (21.07)	83.44 (21.80)
Normal lighting	45	66.29 (22.74)	73.27 (28.16)	75.82 (29.83)

### Measurement of ORF

The STAR norm referenced standardized Early Literacy Test ORF component (McBride et al. 2010) was used to test control and treatment participants under Normal lighting conditions. As mentioned in the background, the Mott et al. (2011) study compared ORF reading scores measured by the standardized AIMSWeb instrument with grade 3 students from middle-income backgrounds. For this study the researchers changed the measurement of ORF towards the STAR protocol because the local school district selected STAR versus AIMSWeb due to the alignment of the reporting-out capabilities of STAR. Although literacy instruction was given under Focus, testing was conducted under Normal lighting conditions for all participants and occurred three times: October, January and May.

STAR tests were administered on the same days in both the experimental and control classes. The exact starting time, between 9 and 10 A.M. was agreed upon and managed by the teachers 9 and 10 A.M. The outdoor conditions during the test days were classified as sunny. The ventilation of the classrooms was controlled and all participating classrooms faced the eastern side of the facility and received the same natural light. A total of 88 students participated in the study.

### Analyses and results

To analyze the ORF differences between the experimental and control conditions, we conducted a 2 (Lighting Type: Normal vs. Focus) by 3 (Time: Beginning vs. Mid-Year vs. End-of-Year) Mixed ANOVA on the Oral Reading Fluency scores with Lighting Type as a between-subjects factor and Time as a within-subjects factor. To follow up the interaction, we first conducted pairwise independent t-tests comparing the Focus and Normal lighting groups within each of the three times. However, none of the t-tests revealed any differences. To validate the effects of Focus during the literacy instruction on the ORF results we therefore conducted separate repeated measures ANOVAs to compare the three times within both the Focus and Normal lighting conditions and conducted six paired-sample t-tests to search for differences among the times within each lighting condition.

There was no main effect of Lighting Type, F(1, 86) = 0.14, MSE = 1529.59, p = .71. while the results show a within-subjects effect of Time, F(2, 172) = 39.42, MSE = 108.79, p < .001. However this main effect was compromised by the Lighting Type by Time interaction effect, F(2, 172) = 5.17, p = .007. Huynh-Feldt adjustments for sphericity were also conducted and supported these results. These findings suggest that although the Focus light setting effect on ORF was insignificant, in order for a significant effect to occur more time in experiencing the ORF instruction during the lighting effect could be required. This led to an analysis of the effect of Time and Focus lighting.

None of the pairwise independent t-tests comparing the Focus and Normal lighting groups within each of the three times revealed any differences. The results showed however differences among the three times (see Table 1) for the both the Normal and Focus lighting groups in the separate repeated measures ANOVAs.

This analysis was conducted to compare the three times within both the Focus and Normal lighting conditions. These results showed that in general, all students performed better on the ORF-test over time indicating the expected learning effect: Grade 3 children are supposed to improve their oral reading fluency over time.

**Figure 1:**
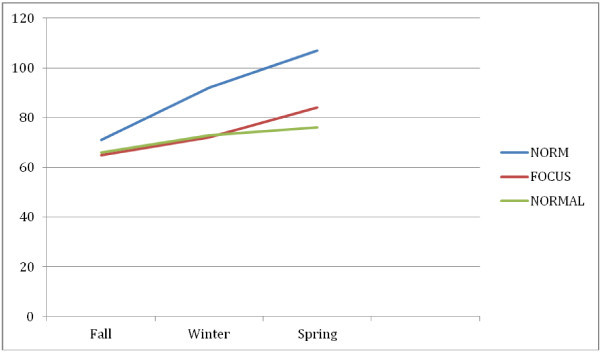
**National oral reading fluency mean scores by focus and normal lighting.** Notes: WCPM column is Words Read Correct Per Minute.

The paired-sample t-tests to search for differences among the times within each lighting condition revealed all statistical differences at data point 3 (83.44 to 73.52).) with an effect size of 0.34, which is between a medium (.25) and large (.40) effect. These findings suggest that the usage of the focus setting during literacy instruction has a positive influence on the gain in oral reading fluency after one year. It is important to note that compared to national ORF norms (see Figure 1), the Focus group students more closely align with the national trend for the upward trajectory by the national sample depicted below.

**Figure 2:**
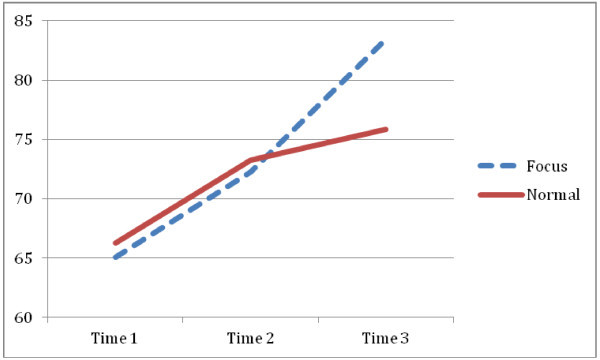
**Means for oral reading fluency scores by lighting condition by time.**

A quasi-experimental research design was used to test the hypothesis that by offering high intensity lighting conditions during the literacy lessons, the uptake of the information will be promoted and as a consequence children will perform better during the examination of ORF. They confirmed that the usage of high intensity yet glare free lighting (referred to as Focus light setting) during reading fluency instruction increased grade 3 students’ oral reading fluency (ORF) scores. The current study further explored the effect of Focus lighting during literacy instruction with at-risk grade 3 students (n = 172) by comparing the ORF gain achieved under when used the oral reading fluency instruction was given under “Focus” lighting with “Normal” lighting. Again the study design allowed for studying an effect of a lighting intervention rather than studying the mechanism why a lighting intervention might support students in their learning behavior. The results of this study confirmed the ORF results measured by the AIMSWEB: Over the course of one academic year, the Focus lighting students increased their ORF scores at a greater rate than did the Normal lighting students. Figure 2 visualizes the words read correct per minute (WCPM) for both the intervention and control group. At the start of the year the ORF scores of the randomly chosen experimental group and the randomly chosen ORF groups were identical confirming that the participating classes did not significantly differ from each other and that in the past they demonstrated similar learning curves. The first half year the scores of the intervention and control group were still similar. In the second half-year, beneficial effects on ORF scores as a result of high intensity lighting during the literacy instructions were measured for the intervention group. They started to outperform their peers in the control classrooms.

This suggests that Focus lighting has educational relevance in bringing at risk students back to the learning progresses that the average student is making, whereas the Normal lighting condition at-risk students remained on a flat trajectory. Therefore, this study demonstrates that the right choice of artificial lighting helps to create an effective learning environment that is crucial to ensuring that at-risk children reach their full potential. Daily literacy instruction under high intensity lighting leads to a higher gain in ORF scores as compared to standard light conditions.

## Conclusion

The State of Mississippi ranks 44 out of 50 in U.S. Department of Education, Institute of Education Sciences, National Center for Education Statistics, National Assessment of Educational Progress (National Center for Education Statistics 2011) rankings for grade 8 student reading ability. The United States ranks close to average in reading compared to 33 other industrialized and developing nations (U.S. Department of Education, Institute of Education Sciences, National Center for EducationStatistics, Program for International Student Assessment PISA 2009). Given these low literacy levels in reading it is incumbent upon educators to seek new methods, materials and technologies for improving reading instruction. The purpose of this study is to evaluate a new lighting technology previously demonstrated to improve cognition and work and school performance. Results incrementally support prior research findings utilizing the AIMSWeb ORF test versus the currently utilized STAR test, indicating evidence for the concurrent validity of score interpretation for implicating focus lighting for improving at-risk student reading performance. Although school performance depends on many parameters, artificial lighting seems to play a key role in helping to create an effective learning environment that is crucial to ensuring that children reach their full potential.

## Endnote

^a^At risk students are defined by a 97% rate of eligibility for free lunch pursuant to the National School Lunch Act. According to Brown (2000). The presence of a high free/reduced lunch percentage exceeding 90% indicates one factor for the presence of at-risk students.
